# miR-672-3p Promotes Functional Recovery in Rats with Contusive Spinal Cord Injury by Inhibiting Ferroptosis Suppressor Protein 1

**DOI:** 10.1155/2022/6041612

**Published:** 2022-02-21

**Authors:** Fang Wang, Jiaxi Li, Yingjie Zhao, Dong Guo, Dongfan Liu, Su'e Chang, Hao Qiao, Jie Li, Yubing Yang, Chengyi Zhang, Rui Wang, Fengtao Li, Dong Wang, Haopeng Li, Xijing He

**Affiliations:** ^1^Department of Orthopaedics, The Second Affiliated Hospital, School of Medicine, Xi'an Jiaotong University, Xi'an, Shaanxi 710004, China; ^2^Department of Orthopaedics, Xi'an International Medical Center Hospital, Xi'an, Shaanxi 710100, China

## Abstract

Aberrantly expressed microRNAs (miRNAs) after spinal cord injury (SCI) participate in diverse biological pathways and processes, including apoptosis, inflammation, oxidative stress responses, peroxidation, and ferroptosis. This study was aimed at exploring the mechanisms underlying miRNA-mediated ferroptosis in an SCI rat model. In the present study, a T10 weight-dropping SCI model was established and miRNA profiling was used to detect miRNA expression profiles post-SCI. Basso-Beattie-Bresnahan scores and inclined plane test, hematoxylin and eosin (HE) and Nissl staining, immunohistochemistry and immunofluorescence, western blotting, cell viability, and Annexin V/7-aminoactinomycin D (7-AAD) assays were used to evaluate locomotor activity, histological changes in the injured spinal cords, neuronal ferroptosis, ferroptosis suppressor protein 1 (FSP1) expression, and cell death, respectively. It was observed that many miRNAs were differentially expressed after SCI, and miR-672-3p, which increased significantly, was selected after cross-referencing with predicted target miRNAs. The luciferase reporter assay demonstrated that miR-672-3p downregulated FSP1, a glutathione-independent ferroptosis suppressor, by binding to its 3′ untranslated region. Oxygen and glucose deprivation- (OGD-) treated PC12 and AGE1.HN cells were treated with miR-672-3p mimics or inhibitors *in vitro*. The effect of miR-672-3p mimics or inhibitor on OGD-PC12/AGE1.HN ferroptosis was evaluated by flow cytometry, immunohistochemistry, immunofluorescence, and western blotting. The miR-672-3p mimics promoted ferroptosis after SCI, whereas the miR-672-3p inhibitor inhibited this process. Rats with SCI treated with miR-672-3p mimics or inhibitor showed similar results *in vivo*. Furthermore, the ferroptosis-related changes caused by SCI or miR-672-3p were reversed by overexpression of FSP1 lentivirus *in vivo* and *in vitro*. These results indicated that sh-miR-672-3p exerted a neural restoration effect *in vivo* and *in vitro* by inhibiting ferroptosis via the FSP1 pathway.

## 1. Introduction

Spinal cord injury (SCI) is a neurological disease that mainly manifests as irreversible functional loss and repair obstruction caused by trauma [1]. The annual incidence of SCI is 12.1–195.4 cases per million worldwide [2]. Many therapeutics have been used to treat patients with SCI, but limited therapeutic opportunities have been demonstrated [3]. Thus, it is necessary to investigate the mechanism underlying the disease pattern and to identify new therapies that have the potential for clinical application.

Ferroptosis has recently been discovered as a type of programmed cell death that is iron-dependent and differs from apoptosis, cell necrosis, and autophagy [4]. Ferroptosis is an iron-dependent form of necrotic cell death that is marked by oxidative damage to phospholipids and can lead to various diseases such as tissue ischemia, reperfusion injury, acute renal failure, and neurodegeneration [5]. The main features of ferroptosis include altered cell morphology and cellular components. In terms of cell morphology, ferroptosis causes cell mitochondria to become smaller, mitochondrial membrane density to increase, and cristae to decrease; however, the morphological changes in the nucleus are not obvious [6]. In terms of cellular components, ferroptosis is manifested by increased lipid peroxidation and reactive oxygen species (ROS) levels. There are also changes in certain characteristic genes [7].

Ferroptosis is thought to be controlled by the phospholipid hydroperoxide-reducing enzyme glutathione peroxidase 4 (GPX4) and radical-trapping antioxidants [8]. However, recent research has reported that the favoprotein apoptosis-inducing factor mitochondria-associated 2 (AIFM2), which is renamed ferroptosis suppressor protein 1 (FSP1), is an antiferroptotic gene. The FSP1-CoQ10-NAD (P) H pathway exists as a stand-alone parallel system that cooperates with GPX4 and glutathione to suppress phospholipid peroxidation and ferroptosis [9, 10]. Ferroptosis is an important biological process during the secondary injury stage after SCI [11]. Previous studies have focused on the association of GPX4 with ferroptosis, and there is still a lack of information regarding the role of the FSP1 pathway in ferroptosis after SCI.

MicroRNAs (miRNAs) control gene expression in key SCI processes, and therapeutic strategies based on miRNAs have attracted increasing interest [12]. In general, miRNAs are small, noncoding RNAs that are 21–25 nucleotides in length and can suppress translation or induce degradation of target mRNAs at the posttranscriptional level by binding to the 3′ untranslated region (UTR) of the gene [13, 14]. Aberrantly expressed miRNAs after SCI participate in diverse biological pathways and processes, including apoptosis, inflammation, oxidative stress responses, peroxidation, and ferroptosis [15, 16]. For example, miR-21 and miR-19b have been reported to be involved in apoptosis and differentiation of neurons [17]; miR-20a, miR-29b, and miR-21 participate in antioxidation and neuroprotection [18, 19]; miR-146a, miR-181, and miR-126 play roles in the inflammatory response [20–22], and miR-21, miR-199a-3p, and miR-9 contribute to axon regeneration and remyelination [23, 24]. However, no study has reported the role of miRNAs in the FSP1 pathway, which is related to ferroptosis after SCI.

In this study, we used miRNA sequencing to select miRNAs that were differentially expressed after SCI. By clustering and functional enrichment analyses of mRNAs, we screened the predicted target genes and ferroptosis-related genes. Then, together with all analyses, we selected miR-672-3p to systematically analyze its effects on functional recovery in rats with contusive spinal cord injury. We also investigated the molecular mechanisms underlying miR-672-3p effects on ferroptosis and oxidative damage. Our results provide new insights into the role of miR-672-3p in ferroptosis *in vivo* and *in vitro* after SCI.

## 2. Materials and Methods

### 2.1. Animals

Adult male Sprague-Dawley rats weighing 200–220 g were purchased from the Animal Center of the Medical Department of Xi'an Jiaotong University and raised in the SPF-level Laboratory Animal Room where the environment was in accordance with standard conditions. This study was approved by the ethics committee of the Second Affiliated Hospital of Xi'an Jiaotong University.

### 2.2. Cell Culture, Transfection, and Oxygen and Glucose Deprivation (OGD) Treatment

The AGE1.HN (ProBioGen AG, Berlin, Germany) and rat pheochromocytoma PC12 cell lines (SCSP-517, Cell Bank of the Chinese Academy of Sciences, Shanghai, China) were cultured in Dulbecco's modified Eagle's medium (DMEM) containing 10% fetal bovine serum (FBS) and 5% horse serum for 2–3 days. To develop an *in vitro* SCI model, the conditions of oxygen and glucose deprivation (OGD) were induced in AGE1.HN and PC12 cells to mimics SCI. The original medium was replaced with glucose-free and phenol-free red DMEM with 10 mmol/L sodium disulfite (Na_2_S_2_O_4_) and deoxidization reagent for 30 min and then returned to the original medium for 24 h until cell damage was detected. Subsequently, the cells were transfected with the miR-672-3p mimics or inhibitor (RiboBio, Guangzhou, China) using Lipofectamine 3000 reagent (Thermo Fisher, USA) or treated with FSP1 lentivirus (GenePharma, Shanghai, China) for 24 h before OGD treatment. The sequences of the mimics or inhibitors used were as follows: sequence for miR-672-3p mimics was 5′-CCGATTCACCAACGA-3′ and the control was 5′-TTTCATACATTCCAGC-3′.

### 2.3. Development of the SCI Model

Consistent with previous studies, the modified Allen method was used to establish SCI models [25]. Briefly, the rats were anesthetized by intraperitoneal injection of 1% pentobarbital sodium (50 mg/kg), and the spines of the rats were fixed. The skin of T9-12 level was incised, and laminectomy was performed at the T10 level to expose the spinal cord. The rats were then placed in the appropriate position of the impactor so that 10 g of rod fell freely at a height of 3 cm and hit the center of the T10 level of the spinal cord. The signs of successful establishment of the model were the appearance of hind limb extension and tail-flick reflex in rats. Laminectomy was performed only in the sham-operated group. The rats in the SCI group received an artificial bladder massage twice a day to assist in urination until they were able to urinate.

### 2.4. Evaluation of Locomotor Capacity

Locomotor capacity was evaluated using the Basso-Beattie-Bresnahan (BBB) exercise scale and the inclined plane test at 1, 3, 5, 7, 10, and 14 days postoperatively. The BBB exercise scale ranges from 0 (complete paralysis) to 21 (normal movement), and it assesses motor function in three parts: joint movement, gait and coordination function, and fine movement of the claws in motion of the hind limbs of the animal. The inclined plane test was performed on an inclined board to measure the maximum angle at which an animal could support its body weight for at least ten seconds without falling. All behavioral tests were performed in a double-blind experiment.

### 2.5. Measurement of Ferroptosis

Ferroptosis was measured using indicators, such as Fe^2+^, GSH, ROS, and malondialdehyde (MDA). To detect iron, spinal cord tissues or cells were immediately homogenized with phosphate-buffered saline (PBS). After centrifugation, the supernatant was tested for iron concentration (Fe^2+^ level) using an iron content determination kit (Abcam, Shanghai, China). To detect GSH, spinal cord tissue or cells were instantly homogenized in 5% trichloroacetic acid and centrifuged at 3,500 rpm for 10 min. The supernatant was used to detect the GSH levels using a GSH assay kit (Nanjing Jiancheng Bioengineering Institute, China). To detect ROS, cells were incubated with 10 *μ*M DCFH-DA probe (Beyotime, Shanghai, China) for 25 min. The cells were then washed twice with PBS, and fluorescence was detected using a fluorescence microplate reader at excitation and emission wavelengths of 488 and 525 nm, respectively. The average fluorescence intensity of each group represented the amount of intracellular ROS. MDA was detected using a commercially available MDA assay kit (BioVision). All kits were used according to the manufacturer's instructions.

### 2.6. Cell Viability Assay

Cell viability was measured using the Cell Counting Kit-8 (CCK-8) assay kit (Biosharp, Hefei, China), according to the manufacturer's instructions. Absorbance at 450 nm was measured using a microplate reader (SpectraMax i3x, Molecular Devices).

### 2.7. Histological Observation of the Spinal Cord

Tissues were fixed in 10% formalin, and following embedding with the embedding agent, 10 *μ*m cross sections were cut at 20°C. The slides were attached and placed at 4°C overnight to dry and then frozen at -20°C for immunofluorescent staining. Hematoxylin and eosin (HE) and Nissl staining kits (Beyotime, Shanghai, China) were used to stain the prepared transverse sections, according to the manufacturer's instructions. Images were then captured using an optical microscope (Nikon ECLIPSE Ti-S, Ruikezhongyi Company). The ratio of the cavity area of the SCI and the number of Nissl bodies were used to evaluate the degree of spinal cord injury.

### 2.8. Western Blotting Assay

FSP1 expression was assessed by western blotting and normalized to that of *β*-actin (1 : 1000, ab9485, Abcam, USA) as a loading control. All proteins in the cells or spinal cord tissues were lysed using RIPA lysis buffer (Beyotime, Shanghai, China). After the protein concentration was determined by BCA protein analysis, 40 *μ*g of total protein was loaded, transferred to a polyvinylidene fluoride (PVDF) membrane (Merck Millipore, Billerica, MA, USA), and separated on 12% SDS-PAGE gels. After blocking with 5% skimmed milk, the membrane was incubated overnight with an indicated primary antibody (1 : 500, LSBio (LifeSpan), AB_1935124) at 4°C. The membranes were then incubated with a horseradish peroxidase-linked IgG secondary antibody (Bioworld, USA) at room temperature for 1 h. The protein bands were exposed to ECL buffer, and signal intensity was measured using ImageJ software. Relative optical density (OD) values of protein bands from western blot analysis were normalized to *β*-actin and determined using a gel imaging analysis system.

### 2.9. miRNA Profiling

Total RNA from rat spinal cord samples from both the SCI and sham groups were extracted using the miRNeasy mini kit (QIAGEN), and miRNA sequencing was used to analyze abnormal miRNA expression. Total RNA was used as input for sequencing library preparation using the TruSeq Small RNA Sample Preparation Kit (Illumina) according to the manufacturer's instructions. Each individual RNA sample had adaptors attached to its 3′ and 5′ ends and was converted into cDNA. The libraries of the two samples were multiplexed at equimolar concentrations and sequenced in two lanes on the Illumina HiSeq 2500 platform, following Illumina's recommended protocol. Quality control was performed on the off-machine raw data, and the process provided by the miRExpress software package was used to trim the adaptor sequences using Trim adapter. A Venn diagram was generated to describe the intersection between the OGD- and ferroptosis-induced miRNA expression, and the expression changes of the selected miRNAs were verified by qRT-PCR.

### 2.10. qRT-PCR Analysis

Total RNA was extracted from spinal cord tissues and cells using TRIzol solution (Invitrogen, Carlsbad, CA, USA). Reverse transcription of extracted RNA was performed using HiScript II Q RT SuperMix (Vazyme, Nanjing, China). The AceQ qPCR SYBR Green Master Mix (Vazyme, Nanjing, China) was used to perform quantitative real-time PCR on an ABI PRISM 7500 Real-Time System. Relative miRNA expression levels were normalized to those of *β*-actin. Primer sequences for miR-672-3p were 5′-CCGATTCACCAACGA-3′, and primer sequences for *β*-actin were F:5′-TGC GTG ACA TTA AGG AGA AG-3′ and R:5′-CAT TGC CGA CAG GAT GCA G-3′.

### 2.11. Cell Death Assay

Cell death was detected using Annexin V/7-aminoactinomycin D (7-AAD) staining. In brief, 5 × 10^5^ cells were resuspended in 100 *μ*L binding buffer and stained with 2.5 *μ*L PE-Annexin V and 2.5 *μ*L 7-AAD at 37°C in darkness for 15 min, and then, 400 *μ*L binding buffer was added. The cells were analyzed by flow cytometry using a fluorescence-activated cell sorting cytometer (BD Biosciences).

### 2.12. TUNEL Assay

The TUNEL assay was used to detect cell death during SCI. For TUNEL detection, the spinal cord tissues containing the damaged areas were embedded in paraffin after being fixed in formalin for 24 h. The TUNEL assay was conducted on 3 mm thick tissue sections using a TUNEL detection kit (Beyotime, Shanghai, China) following the manufacturer's instructions. Nuclei were stained with DAPI (D8200; Solarbio, Beijing, China). The fluorescence intensity of the cells was observed under a Leica DM1000 fluorescence microscope. The positive ratio of the TUNEL assay was used to evaluate cell death between different groups.

### 2.13. Dual-Luciferase Reporter Assay

miRNA target verification was performed in AGE1.HN and PC12 cells using a luciferase reporter assay. Wild-type or mutant FSP1-3UTR dual-luciferase reporter and miR-672-3P mimics, inhibitor, or NC duplexes were cotransfected into AGE1.HN and PC12 cells using Lipofectamine 3000 reagent (Invitrogen), according to the manufacturer's instructions. After 48 h of transfection, the cells were washed with PBS and collected. Luciferase activity was measured using a dual-luciferase reporter assay (Promega, Shanghai, China).

### 2.14. Fluorescence In Situ Hybridization

A fluorescence in situ hybridization (FISH) assay was performed in spinal cord tissues to locate the intracellular position of miR-672-3P. The samples from the SCI and sham groups were fixed with 10% zinc formalin, dehydrated with 70% ethanol, embedded in paraffin, and cut into 5 *μ*m sections for FISH. Hybridization was performed overnight at 50°C using a Dig-labeled miR-672-3P probe. DAPI was used to stain nuclei. Slides were imaged on a Leica DM1000 confocal microscope (Leica Microsystems, Mannheim, Germany) using Olympus cellSens Imaging Software.

### 2.15. Statistical Analyses

All results are presented as the mean ± SEM of experiments performed in triplicate. Student's *t*-test was used for two-group comparisons. Three or more groups of parameters were compared using one-way analysis of variance, and the Bonferroni test was performed on the selected pairs. The data were analyzed using GraphPad Prism 8 statistical software, and *P* < 0.05 was considered statistically significant.

## 3. Results

### 3.1. Ferroptosis Functioned in the Process of SCI *In Vivo*

**Figure 1:**
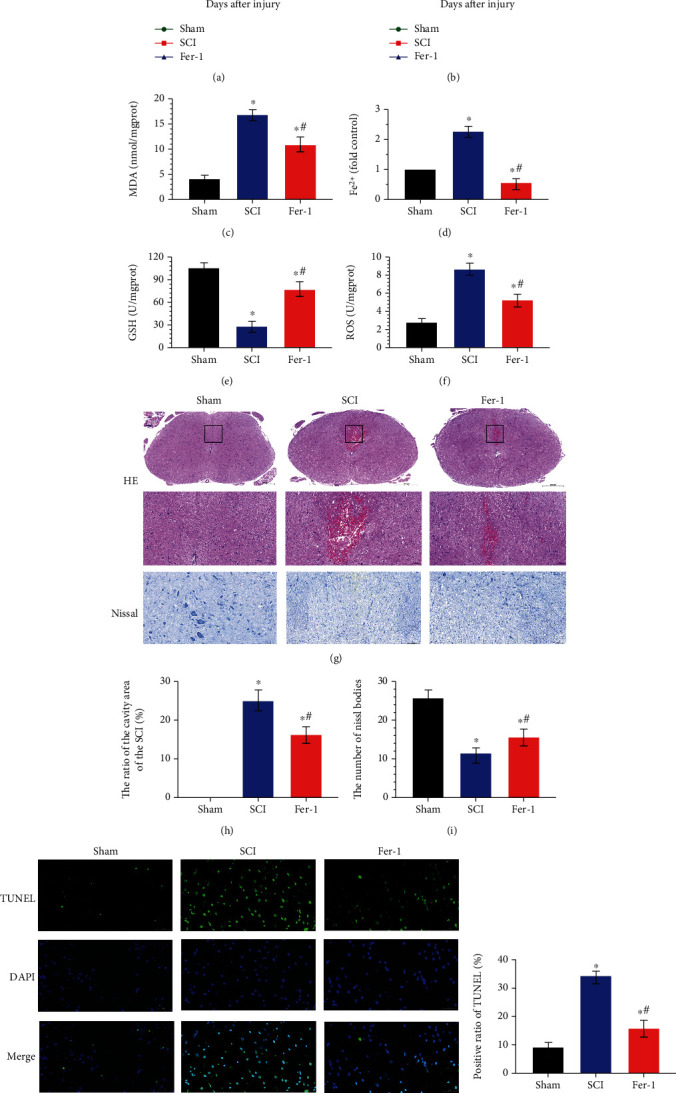
Ferroptosis functioned in the process of SCI in vivo. Sham group: no spinal cord injury or treatment; SCI group: spinal cord injury only; Fer-1 group: Fer-1 treatment after spinal cord injury. (a) Basso-Beattie-Breshman (BBB) exercise scale. (b) Inclined plane test. (c) MDA levels, (d) Fe^2+^ levels, (e) GSH levels, and (f) ROS levels were significantly different between the SCI group and the sham group, and the Fer-1 treatment narrowed the difference. (g) Representative hematoxylin-eosin and Nissl staining of spinal cord sections 7 days after surgery. The neuronal damage in the SCI group was more severe than that in the sham group, while the Fer-1 treatment relieves the damage. (h) Ratio of the area of cavity space to the area of the lesion center 7 days after surgery. (i) Relative number of Nissl bodies of the lesion center 7 days after surgery. (j) Representative TUNEL image of the lesion center 7 days after surgery. (k) Positive ratio of TUNEL in the Fer-1 group was higher than that in the sham group but lower than that in the SCI group. Data are shown as mean ± SD(*n* = 10). ^∗^*P* < 0.05 vs. sham group; ^#^*P* < 0.05 vs. SCI group (one-way analysis of variance followed by the least significant difference test).

To investigate if ferroptosis plays roles in the process of spinal cord injury, rats were divided into three groups: the sham, SCI, and Fer-1 groups. The Fer-1 group was treated with the ferroptosis inhibitor ferrostatin-1 (Fer-1) to determine whether ferroptosis inhibitor can reduce spinal cord injury. The BBB score and inclined plane test were used to evaluate the motor function in rats. As shown in Figures 1(a) and 1(b), the spinal cord injury decreased both BBB score and the angle of incline compared with the sham group, while the ferroptosis inhibitor Fer-1 inhibited the impairment of motor function caused by SCI. MDA, Fe^2+^, ROS, and GSH levels are all regarded as cytological symbols of ferroptosis. HE and Nissl staining was used to evaluate the pathological condition of SCI rats, and the ratio of the cavity area of the SCI and the number of Nissl bodies were regarded as histological symbols to evaluate cell death in the SCI process. A positive TUNEL assay was used to evaluate cell death at the molecular cytological level. The SCI group showed a higher level of MDA, Fe^2+^, and ROS and a lower level of GSH than the sham group; the ratio of the cavity area of the SCI and the positive ratio of TUNEL in the SCI group were significantly higher than those in the sham group; the number of Nissl bodies in the SCI group showed a sharp decrease compared with the sham group, while the treatment with Fer-1 could counteract the ferroptosis-related changes during SCI (Figures 1(c)–1(k)). All these data showed that ferroptosis functioned in the process of SCI, and the ferroptosis inhibitor Fer-1 could mitigate this process *in vivo*.

### 3.2. Ferroptosis Functioned in the Cells Suffering from OGD-Induced Injury *In Vitro*

**Figure 2:**
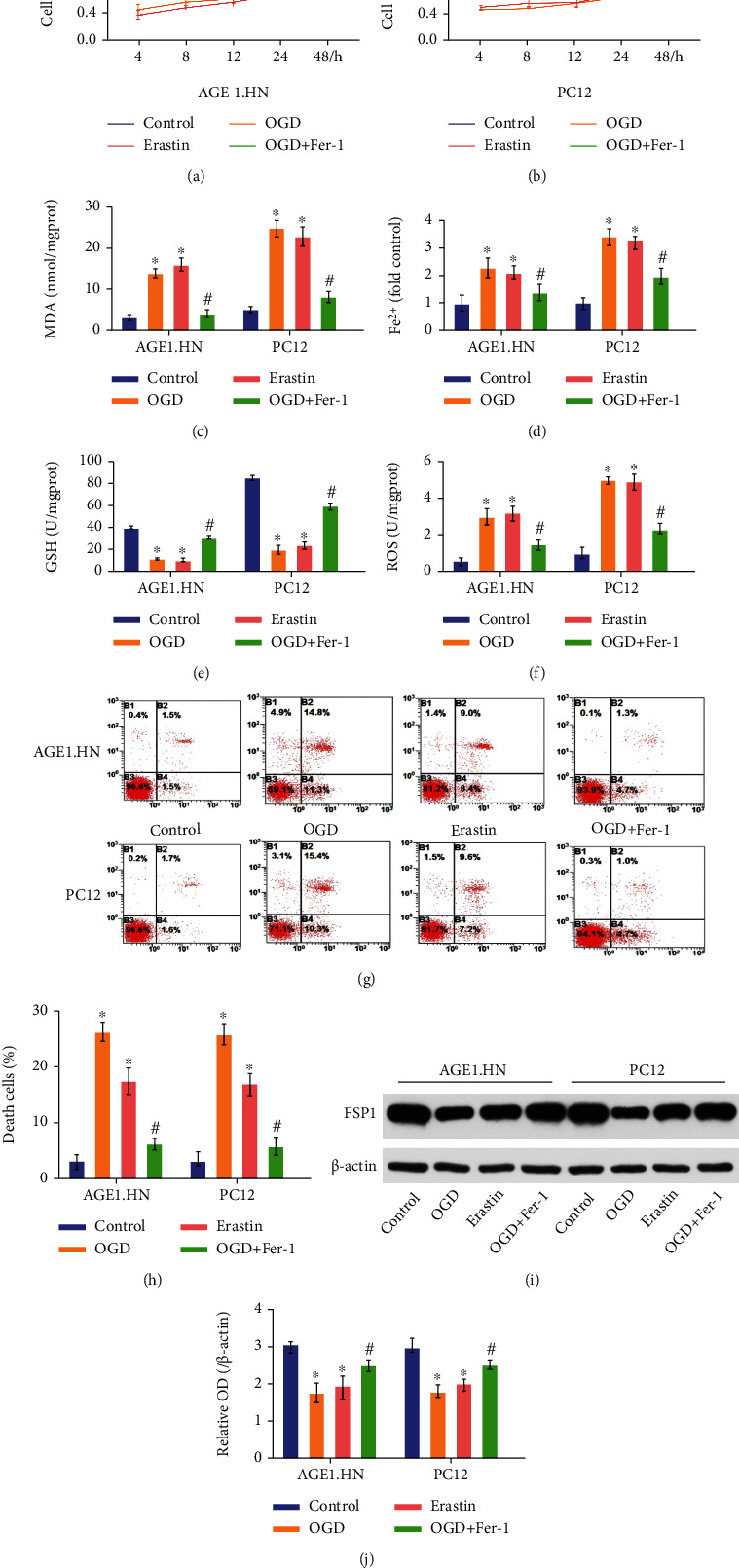
Ferroptosis functioned in the cells suffering from OGD-induced injury in vitro. Control group: no treatment; OGD group: OGD treatment only; erastin group: erastin treatment only; OGD+Fer-1 group: Fer-1 treatment after OGD treatment. (a) Cell viability assay in AGE1.HN cells and in (b) PC12 cells decreased after OGD and erastin treatment, while Fer-1 treatment inhibited the decrease. (c) MDA levels, (d) Fe^2+^ levels, (e) GSH levels, and (f) ROS levels were significantly different between the control group and the OGD/erastin group, and the Fer-1 treatment narrowed the difference. (g) Annexin V/7-AAD assay analyzed by flow cytometry. (h) Death cells increased after OGD and erastin treatment, while Fer-1 treatment inhibited the increase. (i, j) FSP1 expression. Data are shown as mean ± SD(*n* = 10). ^∗^*P* < 0.05 vs. control group; ^#^*P* < 0.05 vs. OGD group (one-way analysis of variance followed by the least significant difference test).

We used a series of cell experiments to verify that ferroptosis also participated in spinal cord injury *in vitro*. Similar to the results of animal experiments, OGD and the ferroptosis inducer erastin treatment caused a typical ferroptosis phenotype of MDA, Fe^2+^, GSH, and ROS levels, which could be alleviated by the ferroptosis inhibitor Fer-1 (Figures 2(c)–2(f)). In addition, OGD and erastin treatment induced cell death in both AGE1.HN and PC12 cells, whereas Fer-1 inhibited OGD-induced cell death, as indicated by the cell viability assay and Annexin V/7-AAD assay (Figures 2(a), 2(b), 2(g), and 2(h)). As confirmed by the previous experiment, FSP1 is a glutathione-independent ferroptosis suppressor [9]. In the OGD and erastin groups, the expression level of FSP1 was suppressed, whereas treatment with Fer-1 inhibited the suppression of FSP1 expression by OGD treatment (Figures 2(i) and 2(j)). These data indicate that ferroptosis is involved in SCI *in vitro* and is related to FSP1 expression.

### 3.3. miR-672-3p Was Aberrantly Expressed during SCI and Targeted FSP1 in Cells

**Figure 3:**
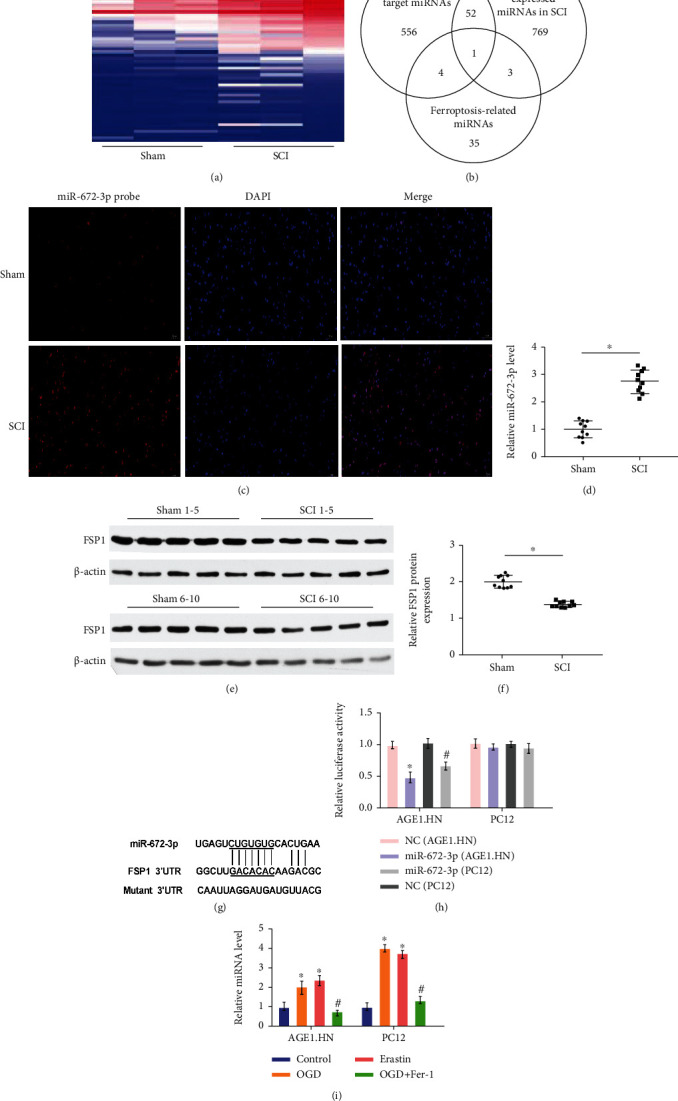
miR-672-3p was aberrantly expressed during SCI and targeted FSP1 in cells. Sham group: no spinal cord injury or treatment; SCI group: spinal cord injury only; control group: no treatment; OGD group: OGD treatment only; erastin group: erastin treatment only; OGD+Fer-1 group: Fer-1 treatment after OGD treatment; NC group: negative analog of miR-672-3p mimics treatment after SCI; miR-672-3p group: miR-672-3p mimics treatment after SCI. (a) Heat map showing expression profiles for the sham group compared with the SCI group. (b) Venn diagram showing the intersection of differentially expressed miRNAs in SCI, predicted target miRNAs, and ferroptosis-related miRNAs. (c) FISH showing the expression of miR-672-3p increased significantly in the SCI group compared with the sham group. (d) Relative miR-672-3p level (^∗^*P* < 0.05 vs. sham group). (e, f) FSP1 expression level decreased in the SCI group compared with the sham group (^∗^*P* < 0.05 vs. sham group). (g) The predicted binding site between miR-672-3p and 3′-UTR of FSP1 mRNA. (h) Relative luciferase activity in AGE1.HN and PC12 cells cotransfected with miR-672-3p with wild-type FSP1 luciferase (^∗^*P* < 0.05 vs. NC (AGE1.HN) group; ^#^*P* < 0.05 vs. NC (PC12) group). (i) Relative miRNA level (^∗^*P* < 0.05 vs. control group; ^#^*P* < 0.05 vs. OGD group). Data are shown as mean ± SD(*n* = 10) (one-way analysis of variance followed by the least significant difference test).

To further investigate the function of miRNAs in the regulation of ferroptosis during SCI, we screened the miRNAs that were aberrantly expressed in the SCI and sham groups by miRNA profiling (Figure 3(a)). We identified 769 miRNAs that were differentially expressed during SCI and 35 miRNAs related to ferroptosis (Figure 3(b)). Cross-referencing with predicted target miRNAs, we selected miR-672-3p, which increased significantly in the SCI group compared to the sham group (Figures 3(c) and 3(d)). Furthermore, miR-672-3p significantly increased in OGD-induced and erastin-treated AGE1.HN and PC12 cells, whereas Fer-1 decreased miRNA levels in OGD-induced AGE1.HN and PC12 cells *in vitro* (Figure 3(i)). In contrast, the expression of FSP1 in the SCI group was significantly higher than that in the SCI group and was negatively correlated with the expression of miR-672-3p (Figures 3(e) and 3(f)). To further investigate the regulatory relationship between miR-672-3p and FSP1, we performed a bioinformatics assay and found that FSP1 was a potential target gene of miR-672-3p (Figure 3(g)). We transfected miR-672-3p mimics with the wild-type FSP1 luciferase reporter vector into AGE1.HN and PC12 cells and detected the luciferase activity. As shown in Figure 3(h), cotransfection of miR-672-3p with wild-type FSP1 luciferase significantly decreased luciferase activity compared to the mutant FSP1 luciferase reporter, indicating that miR-672-3p directly binds to the 3′-UTR of FSP1. These data indicate that miR-672-3p was aberrantly expressed during SCI and targeted FSP1 in cells.

### 3.4. miR-672-3p Promotes Ferroptosis during SCI

**Figure 4:**
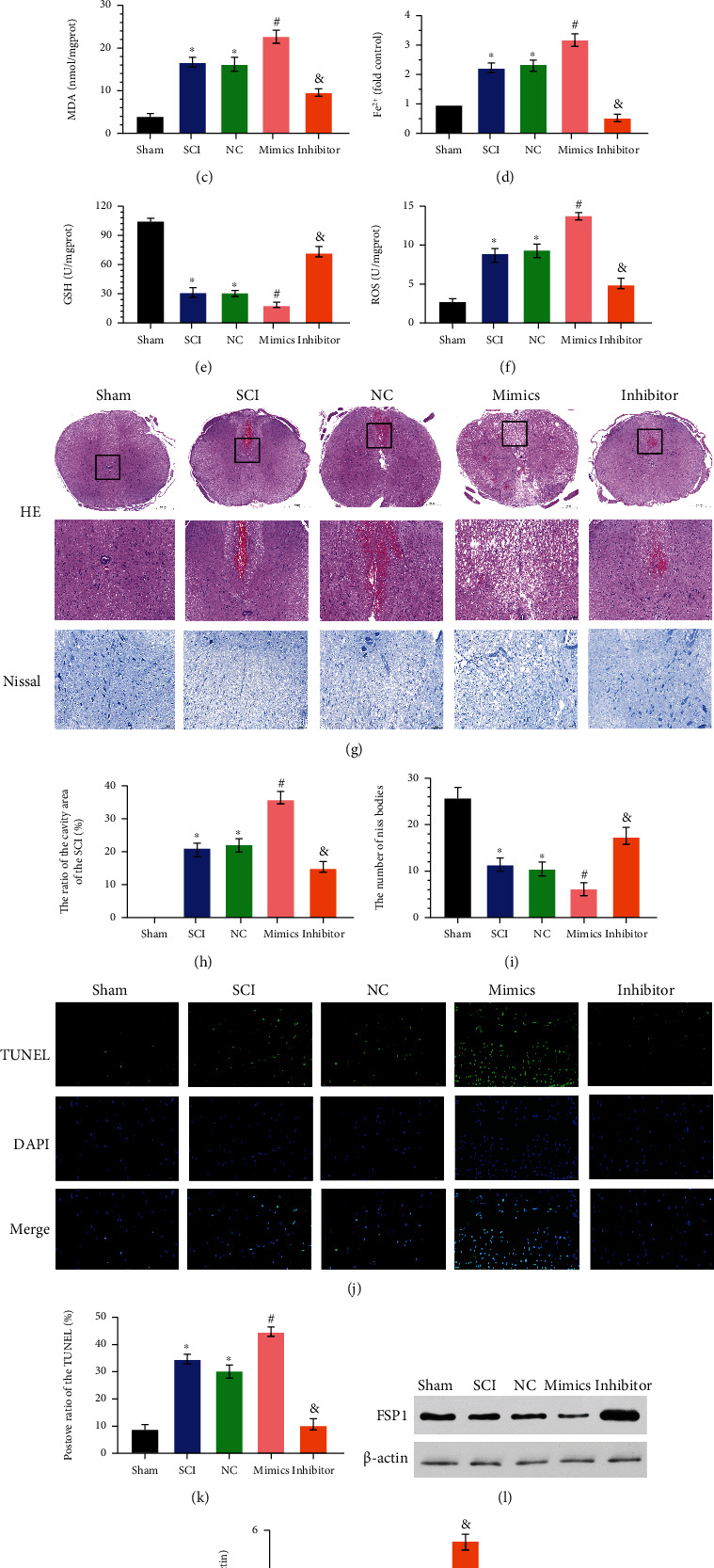
miR-672-3p promotes ferroptosis during SCI. Sham group: no spinal cord injury or treatment; SCI group: spinal cord injury only; NC group: negative analog of miR-672-3p mimics treatment after SCI; miR-672-3p mimics group: miR-672-3p mimics treatment after SCI; miR-672-3p inhibitor group: miR-672-3p inhibitor treatment after SCI. (a) Basso-Beattie-Breshman (BBB) exercise scale. (b) Inclined plane test. (c) MDA levels, (d) Fe^2+^ levels, (e) GSH levels, and (f) ROS levels. (g) Representative hematoxylin-eosin and Nissl staining of spinal cord sections 7 days after surgery. The miR-672-3p mimics group increased the neuronal damage while the miR-672-3p inhibitor group decreased it. (h) Ratio of the area of cavity space to the area of the lesion center 7 days after surgery. (i) Relative number of Nissl bodies of the lesion center 7 days after surgery. (j) Representative TUNEL image of the lesion center 7 days after surgery. (k) Positive ratio of TUNEL in the miR-672-3p mimics group increased while that in the miR-672-3p inhibitor group decreased compared with that in the SCI group. (l, m) The miR-672-3p mimics downregulated FSP1 expression while the miR-672-3p inhibitor upregulated it. Data are shown as mean ± SD(*n* = 10). ^∗^*P* < 0.05 vs. sham group; ^#,*&*^*P* < 0.05 vs. SCI group (one-way analysis of variance followed by the least significant difference test).

To further investigate the regulatory effects of miR-672-3p on ferroptosis, rats were divided into five different groups: the sham, SCI, SCI+NC (NC), SCI+miR-672-3p mimics (mimics), and SCI+miR-672-3p inhibitor groups (inhibitor). The mimics group showed impairment of motor function caused by SCI, while the inhibitor group showed inhibited impairment of motor function (Figures 4(a) and 4(b)). The MDA levels increased significantly in the mimics group and decreased in the inhibitor group (Figure 4(c)). The Fe^2+^ and ROS levels showed the same trend as MDA in the different groups (Figures 4(d) and 4(f)). The GSH levels increased significantly in the inhibitor group and decreased in the mimics group (Figure 4(e)). The mimics group showed an increased ratio of the cavity area of the SCI, while the inhibitor group reduced it (Figures 4(g) and 4(h)). The number of Nissl bodies decreased in the mimics group and increased in the inhibitor group compared with that in the NC group (Figures 4(g) and 4(i)). The positive TUNEL ratio indicated that the miR-672-3p mimics promoted cell death during SCI, while the miR-672-3p inhibitor inhibited this process (Figures 4(j) and 4(k)). The miR-672-3p mimics also promoted the expression of FSP1, while the miR-672-3p inhibitor inhibited it (Figures 4(l) and 4(m)). These data suggest that miR-672-3p promotes ferroptosis during SCI by inhibiting the expression of FSP1.

### 3.5. miR-672-3p Promotes Ferroptosis in the OGD-Induced Cells

**Figure 5:**
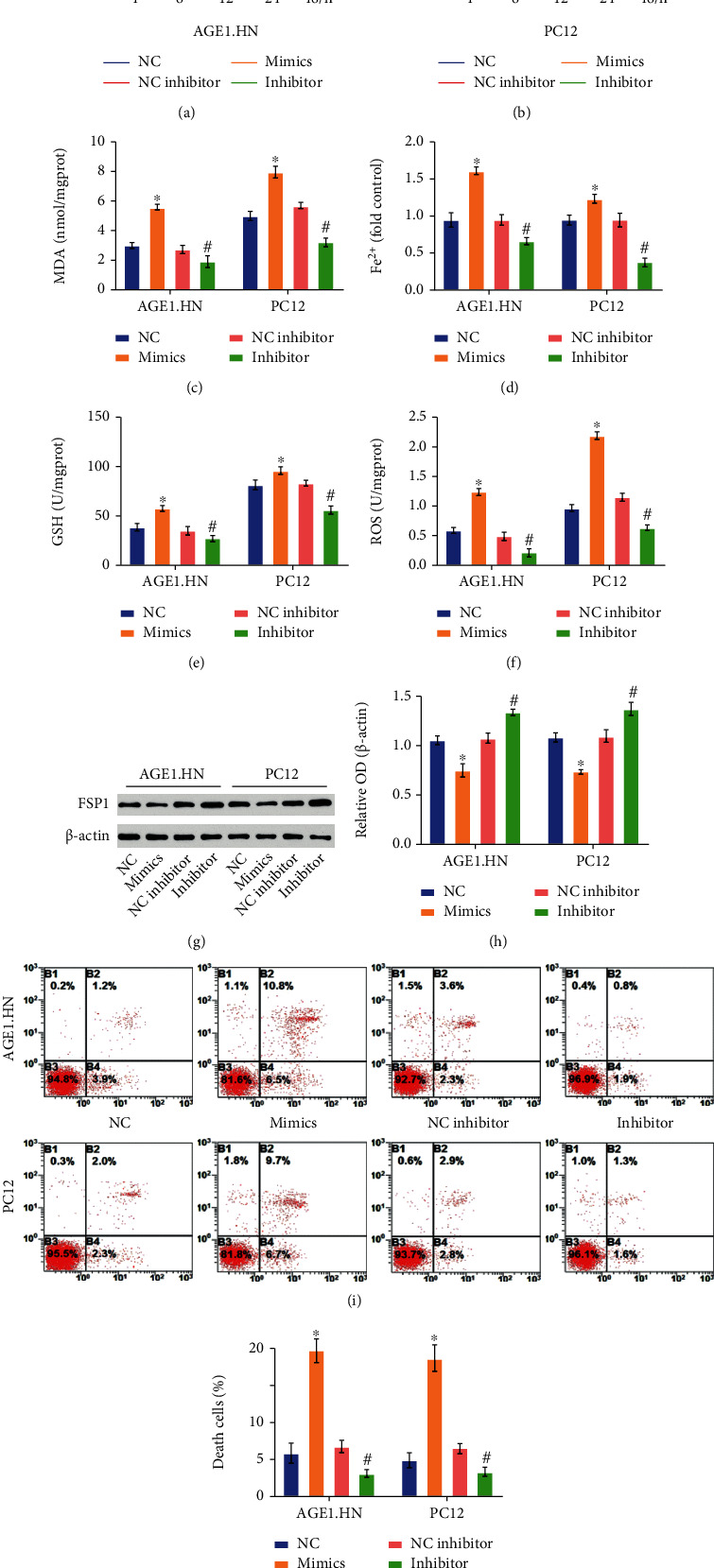
miR-672-3p promotes ferroptosis in the OGD-induced cells. NC group: negative analog of miR-672-3p mimics treatment after OGD; NC inhibitor: negative analog of miR-672-3p inhibitor treatment after OGD; mimics group: miR-672-3p mimics treatment after OGD; inhibitor group: miR-672-3p inhibitor treatment after OGD. (a) Cell viability assay in AGE1.HN cells and in (b) PC12 cells decreased after miR-672-3p mimics treatment and increased after miR-672-3p inhibitor treatment. (c) MDA levels, (d) Fe^2+^ levels, (e) GSH levels, and (f) ROS levels. (g, h) FSP1 expression. (i) Annexin V/7-AAD assay analyzed by flow cytometry. (j) Death cells showed the same trend as the cell viability assay. Data are shown as mean ± SD(*n* = 10). ^∗^*P* < 0.05 vs. NC group; ^#^*P* < 0.05 vs. NC inhibitor group (one-way analysis of variance followed by the least significant difference test).

Cell experiments were performed to verify that miR-672-3p regulated ferroptosis by targeting FSP1 *in vitro*. We used the mimics and inhibitor of miR-672-3p to upregulate and downregulate miRNAs, respectively, as described above. In both AGE1.HN and PC12 cells, the miRNA mimics enhanced the increase in MDA, Fe^2+^, and ROS levels caused by OGD-induced injury, whereas the miRNA inhibitor decreased it (Figures 5(c), 5(d), and 5(f)). The miRNA mimics lowered GSH levels, while the miRNA inhibitor increased them (Figure 5(e)). Cell death was measured by the cell viability assay and Annexin V/7-AAD assay, and the results of both assays showed that the miRNA mimics promoted cell death in ferroptosis, while the miRNA inhibitor prevented this process (Figures 5(a), 5(b), 5(i), and 5(j)). Changes in these indicators proved that miR-672-3p promoted ferroptosis in OGD-induced AGE1.HN and PC12 cells. The western blot assay indicated that the expression of FSP1 was downregulated by the miRNA mimics and upregulated by the miRNA inhibitor, which strongly supports that miR-672-3p promotes ferroptosis in OGD-induced cells by targeting FSP1.

### 3.6. Overexpression of FSP1 Inhibits Ferroptosis during SCI

**Figure 6:**
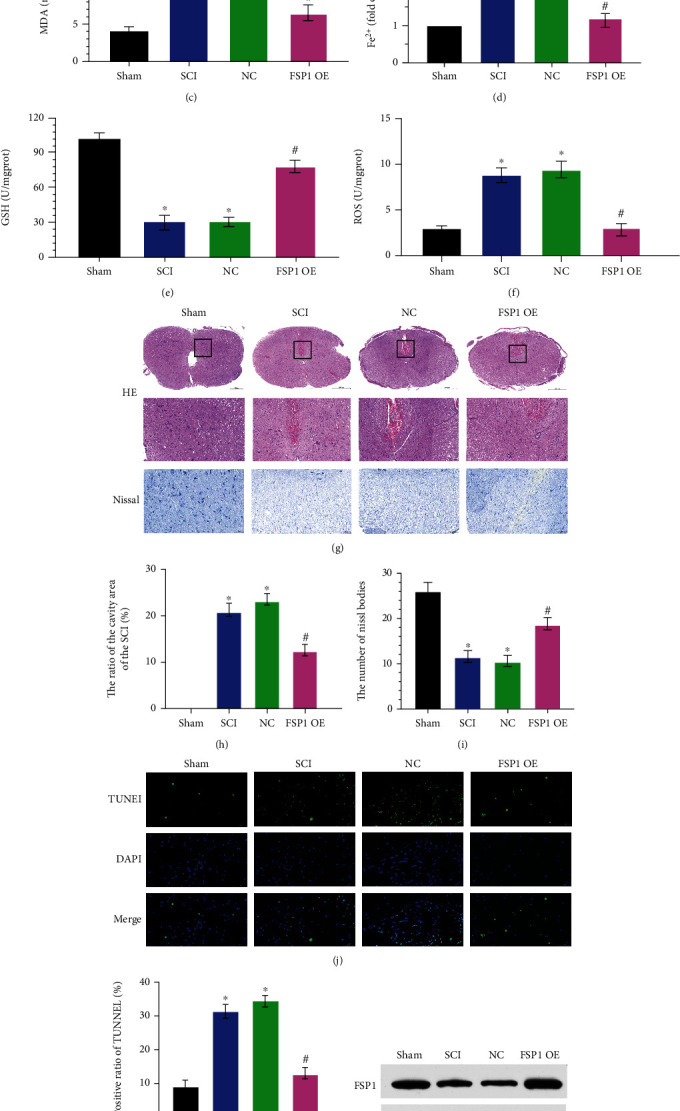
The overexpression of FSP1 inhibits ferroptosis during SCI. Sham group: no spinal cord injury or treatment; SCI group: spinal cord injury only; NC group: negative analog of overexpression FSP1 lentivirus treatment after SCI; FSP1 OE group: overexpression FSP1 lentivirus treatment after SCI. (a) Basso-Beattie-Breshman (BBB) exercise scale. (b) Inclined plane test. (c) MDA levels, (d) Fe^2+^ levels, (e) GSH levels, and (f) ROS levels. (g) Representative hematoxylin-eosin and Nissl staining of spinal cord sections 7 days after surgery. The FSP1 OE group decreased the neuronal damage. (h) Ratio of the area of cavity space to the area of the lesion center 7 days after surgery. (i) Relative number of Nissl bodies of the lesion center 7 days after surgery. (j) Representative TUNEL image of the lesion center 7 days after surgery. (k) Positive ratio of TUNEL in the FSP1 OE group decreased compared with the SCI and NC groups. (l, m) The overexpression FSP1 lentivirus treatment upregulated FSP1 expression. Data are shown as mean ± SD(*n* = 10). ^∗^*P* < 0.05 vs. sham group; ^#^*P* < 0.05 vs. SCI group (one-way analysis of variance followed by the least significant difference test).

To verify the regulatory role of FSP1 in the process of ferroptosis during SCI, SCI rats were treated with lentivirus overexpressing FSP1. Western blotting indicated that the overexpression of FSP1 lentivirus treatment inhibited SCI-induced downregulation of FSP1 expression (Figures 6(l) and 6(m)). As shown in Figures 6(a) and 6(b), the improvement in the BBB score and angle of inclination indicates that overexpression of FSP1 promotes the recovery of motor function after spinal cord injury. Changes in indicators such as MDA, GSH, Fe^2+^, and ROS also suggest that overexpression of FSP1 inhibits ferroptosis during SCI at the molecular level (Figures 6(c)–6(f)). Overexpression of FSP1 scaled the ratio of the cavity area of the SCI down and increased the number of Nissl bodies compared to the NC and SCI groups (Figures 6(g)–6(i)). The significant decrease in the positive ratio of TUNEL indicated that overexpression of FSP1 inhibited cell death during SCI (Figures 6(j) and 6(k)). These data support the hypothesis that the overexpression of FSP1 inhibits ferroptosis during SCI.

### 3.7. miR-672-3p Promotes Ferroptosis during SCI via Downregulation of FSP1

**Figure 7:**
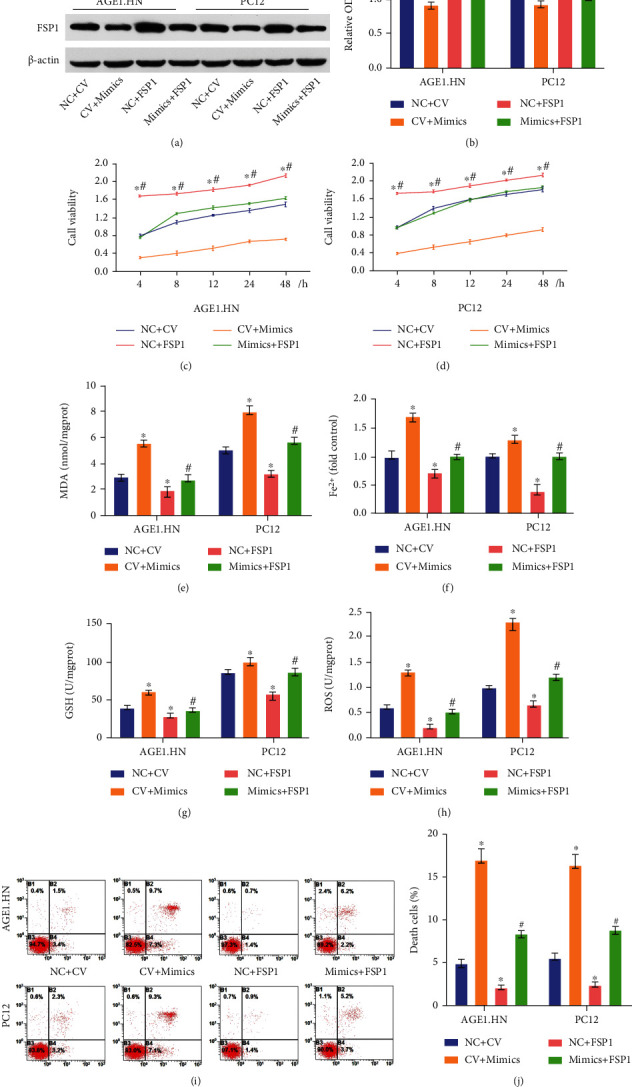
miR-672-3p promotes ferroptosis during SCI by downregulating the expression of FSP1. NC+CV group: negative analog of overexpression FSP1 lentivirus and miR-672-3p mimics treatment; NC+FSP1 group: overexpression FSP1 lentivirus and negative analog of miR-672-3p mimics treatment; CV+mimics group: miR-672-3p mimics and negative analog of overexpression FSP1 lentivirus; mimics+FSP1 group: overexpression FSP1 lentivirus and miR-672-3p mimics treatment. (a, b) FSP1 expression increased after overexpression FSP1 lentivirus treatment. The overexpression of FSP1 upregulated the cell viability assay in (c) AGE1.HN cells and in (d) PC12 cells after miR-672-3p mimics treatment. (e) MDA levels, (f) Fe^2+^ levels, (g) GSH levels, and (h) ROS levels. (i) Annexin V/7-AAD assay analyzed by flow cytometry. (j) Death cells showed the same trend as the cell viability assay. Data are shown as mean ± SD(*n* = 10). ^∗^*P* < 0.05 vs. NC+CV group; ^#^*P* < 0.05 vs. CV+mimics group (one-way analysis of variance followed by the least significant difference test).

To evaluate whether miR-672-3p regulates ferroptosis during SCI by targeting FSP1, we treated OGD-treated AGE1.HN and PC12 cells with miR-672-3p mimics, FSP1 lentivirus, cotreatment with miR-672-3p mimics, and overexpression of FSP1 lentivirus. Western blotting indicated that the overexpression of FSP1 by lentivirus treatment upregulated the miRNA-induced downregulation of FSP1 expression (Figures 7(a) and 7(b)). To verify the regulation of ferroptosis, MDA, Fe^2+^, ROS, GSH levels, and cell death were detected as previously mentioned. As shown in Figures 7(e)–7(h), the miR-672-3p mimics treatment increased the levels of MDA, Fe^2+^, and ROS and decreased the level of GSH, which could be inhibited by the overexpression of FSP1. The cell viability assay and Annexin V/7-AAD assay both showed that the overexpression of FSP1 reduced the rate of cell death caused by miR-672-3p (Figures 7(c), 7(d), 7(i), and 7(j)). These data proved that miR-672-3p promotes ferroptosis during SCI by downregulating the expression of FSP1.

## 4. Discussion

Ferroptosis is a newly discovered form of programmed cell death that occurs due to accumulation of iron-dependent lipid peroxide; the term “ferroptosis” was first used by Dixon et al. in 2012 [26]. Ferroptosis is genetically, morphologically, and biochemically different from apoptosis [27] and is characterized by an iron-dependent increase in ROS, which plays a critical role in ferroptosis [28]. Ferroptosis is considered to be involved in the pathological cell death associated with Alzheimer's [29], Huntington's [30], and Parkinson's diseases [31], stroke, traumatic brain injury, ischemia-reperfusion injury, and others [32]. In recent years, an increasing number of studies have focused on the role of ferroptosis in SCI [33–35].

In this study, we found that after SCI, the expression levels of ferroptosis markers in the spinal cord tissue of SCI rats were obviously changed; for example, Fe^2+^ increased and GSH decreased, indicating that ferroptosis occurred after SCI. MDA and ROS levels increased after SCI. MDA activity can directly reflect the level of oxygen free radicals in the body and indirectly reflect the degree of damage to tissues or cells by free radicals [36]. ROS plays a critical role in ferroptosis [28]. These findings are consistent with those of previous studies [37–39]. Furthermore, the expression levels of all the ferroptosis markers mentioned above after SCI could be rescued by Fer-1 intervention. In particular, the effects of functional recovery in rats with contusive spinal cord injury were improved after Fer-1 intervention compared with the control group. This curative effect may be related to the histological basis that Fer-1 reduces damage to nerve cells and tissues and promotes functional recovery. By observing the morphology of the SCI site after using Fer-1, it was found that the neuron structure of degeneration and edema in HE gradually became clear and the nucleus was clearly visible. Further exploration of the changes in the structure of the Nissl body in the neurons revealed that the Nissl body in the SCI group swelled after injury and was elliptical, irregular, or dumbbell-shaped with cavitation. In the Fer-1 treatment group, the cavitation of the Nissl body was also reduced compared with that in the SCI group, and the structure of the Nissl body began to become complete. TUNEL results also indicated that cell death decreased in the Fer-1 treatment group. These results show that ferroptosis may play an important role in the serious consequences of secondary injury following SCI and that regulating this process may facilitate functional recovery after SCI.

In 2012, Cell Magazine proposed a new type of programmed cell death, ferroptosis. This is a method of cell death caused by iron-dependent lipid peroxidation, which degrades cell membranes [26]. Previous studies have suggested that GPX4 is the main regulator of ferroptosis [40, 41]. Knockout of GPX4 causes accumulation of reactive oxygen free radicals on membrane lipids and induces ferroptosis. However, in 2019, a study proved that the FSP1-CoQ10-NAD(P)H pathway exists as a stand-alone parallel system that cooperates with GPX4 and glutathione to suppress phospholipid peroxidation and ferroptosis [9]. Our study showed that FSP1 expression decreased after SCI in both *in vivo* and *in vitro* experiments. This situation could be aggravated by erastin and rescued by Fer-1. These findings indicated for the first time that FSP1 is a potential regulator of ferroptosis during secondary injury following SCI.

SCI causes substantial miRNA changes [42], and miRNAs regulate the protein expression of their target genes at the posttranslational level, leading to cascading pathophysiological events, including inflammatory and immune activation, excitotoxicity, oxidative stress, and neuronal activity imbalances [43, 44]. However, few miRNA studies of SCI have focused on ferroptosis. A single miRNA can regulate hundreds of mRNAs and control the expression of many genes that participate in functional interaction pathways. In this study, we used miRNA sequencing to select miRNAs that were differentially expressed after SCI. miRanda (http://www.microrna.org) and TargetScan (http://www.targetscan.org) were used to identify targets of significantly differentially expressed miRNAs. In particular, we focused on the target gene, FSP1, which is a potential new regulator of ferroptosis. Finally, we selected miR-672-3p, which was highly expressed after SCI and may target FSP1. Through *in vivo* and *in vitro* experiments, we demonstrated that miR-672-3p could suppress the expression of FSP1. This was a new finding that proved to be a new therapeutic method for SCI.

To further investigate the relationship between miR-672-3p and ferroptosis, along with recovery effects in rats with contusive spinal cord injury, we designed both animal- and cell-based experiments. By enhancing and inhibiting the expression of miR-672-3p, we found that the ferroptosis process was aggravated and the effects of functional recovery were attenuated after miR-672-3p was enhanced. In contrast, ferroptosis was suppressed, and the effects of functional recovery were improved after miR-672-3p inhibition. More importantly, FSP1 played a pivotal role in all above biological processes, and overexpression of FSP1 suppressed ferroptosis and increased the effects of neural restoration, thereby improving functional recovery in rats with contusive spinal cord injury. On the other hand, the low expression of FSP1 not only aggravated the ferroptosis process and attenuated the effects of recovery level of SCI but also reversed the repair effects of miR-672-3p overexpression. Through our research, we found a new “miR-672-3p-FSP1-ferroptosis-functional recovery of SCI” pathway that revealed the contribution of ferroptosis to neuronal demise and glial scar formation, which may provide a new therapeutic opportunity for SCI.

## 5. Conclusions

miR-672-3p exerts a neural restoration effect *in vivo* and *in vitro* by inhibiting ferroptosis via the FSP1 pathway. In addition, miR-672-3p improved locomotor function in SCI rats, suggesting its potential as a target for the development of therapeutics for SCI.

## Data Availability

The raw data of experiments used to support the findings of this study are available from the corresponding authors upon request.
